# Sustainable Microextraction Using Switchable Solubility Solvent for the Liquid Chromatographic Determination of Three Profenoid Drugs in Urine Samples

**DOI:** 10.1002/jssc.70223

**Published:** 2025-07-12

**Authors:** Christina Patakidou, Marianna Ntorkou, Constantinos K. Zacharis

**Affiliations:** ^1^ Laboratory of Pharmaceutical Analysis Department of Pharmacy Aristotle University of Thessaloniki Thessaloniki Greece; ^2^ Laboratory of Analytical Chemistry Department of Chemistry Aristotle University of Thessaloniki Thessaloniki Greece

**Keywords:** HPLC, microextraction, non‐steroidal anti‐inflammatory drug, switchable solubility solvent, urine

## Abstract

Herein, a switchable solubility solvent (sodium salicylate) was employed for the microextraction of “profenoid” drugs (i.e., ketoprofen, fenoprofen, and flurbiprofen) from human urine samples. This approach utilizes the phase transition of salicylic acid (0.75 mol/L) by altering the sample's pH (addition of 10 µL of H_3_PO_4_ 10 M), facilitating efficient dispersion and phase separation in a single step. The solidified salicylic acid was easily collected using a syringe filter (nylon). In addition, the extensive contact area between the solidified solvent and the sample solution ensured effective extraction of the target analyte. The critical experimental parameters affecting the extraction performance of the analytes were examined. The separation of the drugs was carried out on a C_18_ analytical column using gradient elution. Excellent linearity was observed in the dynamic range of 50–3000 ng/mL while the precision (%RSD) was less than 14.3% in all cases. The intraday and interday trueness were satisfactory, being in the range of 82.3%–110.1%. The green potential of the proposed analytical scheme was examined based on AGREEprep, ComplexMoGAPI, CACI, and AGREE metric tools. The developed method was utilized for the analysis of authentic urine samples after oral administration of a ketoprofen‐containing formulation.

## 1 Introduction

“Profenoid” drugs including ketoprofen (KTP), fenoprofen (FNP), and flurbiprofen (FRP) belong to the non‐steroidal anti‐inflammatory drugs (NSAIDs) which are commonly used to relieve pain, reduce fever and inflammation, and lower the risk of blood clots. Their action is based on the inhibition of two known isoenzymes of prostaglandin G/H synthase commonly referred to as cyclooxygenases [1]. Although profenoid drugs are typically regarded as safe, they may lead to serious adverse effects such as gastrointestinal bleeding, intestinal ulcers, aplastic anemia, and reduced platelet aggregation. These potential risks underscore the importance of developing selective and sensitive analytical methods for the determination of these drugs in biological fluids.

Microextraction techniques are receiving growing attention, as numerous protocols have been introduced and applied in bioanalysis. Various analytical protocols have been developed to enhance the sensitivity, extraction performance, and environmental sustainability of microextraction procedures [2]. These innovations include the use of novel sorbent materials in small quantities for sorbent‐based microextraction and the application of green solvents in liquid‐phase microextraction (LPME) [3, 4]. The use of micro‐scale adsorbents and eco‐friendly solvents significantly alleviates the limitations of microextraction techniques. In addition, multi‐phase extraction protocols have been proposed that combine two preconcentration techniques, such as solid phase microextraction and LPME, to enhance analyte recovery and selectivity [5]. This hybrid approach improves the sensitivity and trueness of the method, allowing for the analysis of a broader range of chemicals in a single sample.

Switchable hydrophilicity solvents (SHSs) have become a growing trend in the microextraction field over the past decade [6, 7, 8, 9, 10, 11, 12]. These solvents can be reversibly transformed between different states, each with distinct physicochemical properties such as hydrophilicity, solubility, and so on. They may be amines, amidines, and fatty acids that can alter their phase state and chemical structure under specific conditions. In addition, various hydrophilic solvents that can alter their phase state and chemical structure through controlled factors such as pH, temperature, metathesis reactions, organic solvents, and the salting‐out effects [7]. These modifications enhance extraction efficiency and create a larger contact surface area. For instance, the polarity of amine‐based SHSs can be adjusted by introducing or removing carbon dioxide which makes them either water‐miscible or water‐immiscible. In terms of environmental impact, SHSs are considered safer than traditional organic solvents (e.g., halogenated solvents) and their use in analytical chemistry is supported in numerous reports [13].

In the last years, various analytical protocols for the determination of profenoid drugs in biological samples have been reported including fabric phase sorptive extraction (FPSE) [14, 15], covalent organic framework (COF) [16], metal organic framework (MOF) [17], hollow fiber (HF) membrane‐based liquid phase microextraction [18], automated electric‐field‐driven LPME [19], solid phase extraction (SPE) [20, 21], single drop microextraction (SDME) [22], and microextraction by packed sorbent [23]. To the best of our knowledge, only one method has been developed for the determination of KTP and FRP that utilizes SHS in liquid–liquid phase microextraction [24]. Potential drawbacks of this approach include the usage of a relatively high volume of organic solvents involving acetonitrile (4 mL) and *N*,*N*‐dimethylcyclohexylamine (extraction solvent, 500 µL).

In this study, we introduce a novel green liquid phase microextraction technique that uses salicylic acid as switchable solubility solvent (SSS) for the extraction and quantification of KTP, FNP, and FRP in human urine samples. The extraction process involves adding sodium salicylate to the aqueous sample, followed by the in situ solidification of the extractant under acidic conditions. This analytical method enables rapid dispersion of the solvent in the aqueous phase without requiring any external equipment. The factors influencing the efficiency of the microextraction process (i.e, type and concentration, sample ionic strength, acid type and concentration) have been examined. The greenness evaluation of the method has been performed using the AGREEprep and ComplexMoGAPI tools. This analytical approach was applied to determine the analytes in authentic human urine samples after administration of KTP‐containing pharmaceutical formulation.

## 2 Experimental

### 2.1 Reagents and Solutions

All drugs (KTP, FNP, and FRP) and ibuprofen (used as internal standard [ISTD]) (Figure S1), methanol (MeOH, HPLC grade), sodium salts of salicylic acid, benzoic acid, sorbic acid, and phthalic acid were of analytical grade (≥ 98.0%) and provided by Merck (Darmstadt, Germany). Concentrated H_3_PO_4_ (85%), HCl (37% w/w), HCOOH (≥ 99.0%), and glacial CH_3_COOH (≥ 98.0%) were purchased by Sigma–Aldrich (St. Louis, MO, USA). Ultrapure water was produced by a B30 purification system (Adrona SIA, Riga, Latvia).

Stock standard solution (1000 µg/mL) of each drug were individually prepared in MeOH and were kept at 4°C. Working standards were made in water from the stock solutions.

### 2.2 Microextraction Procedure

**FIGURE 1:**
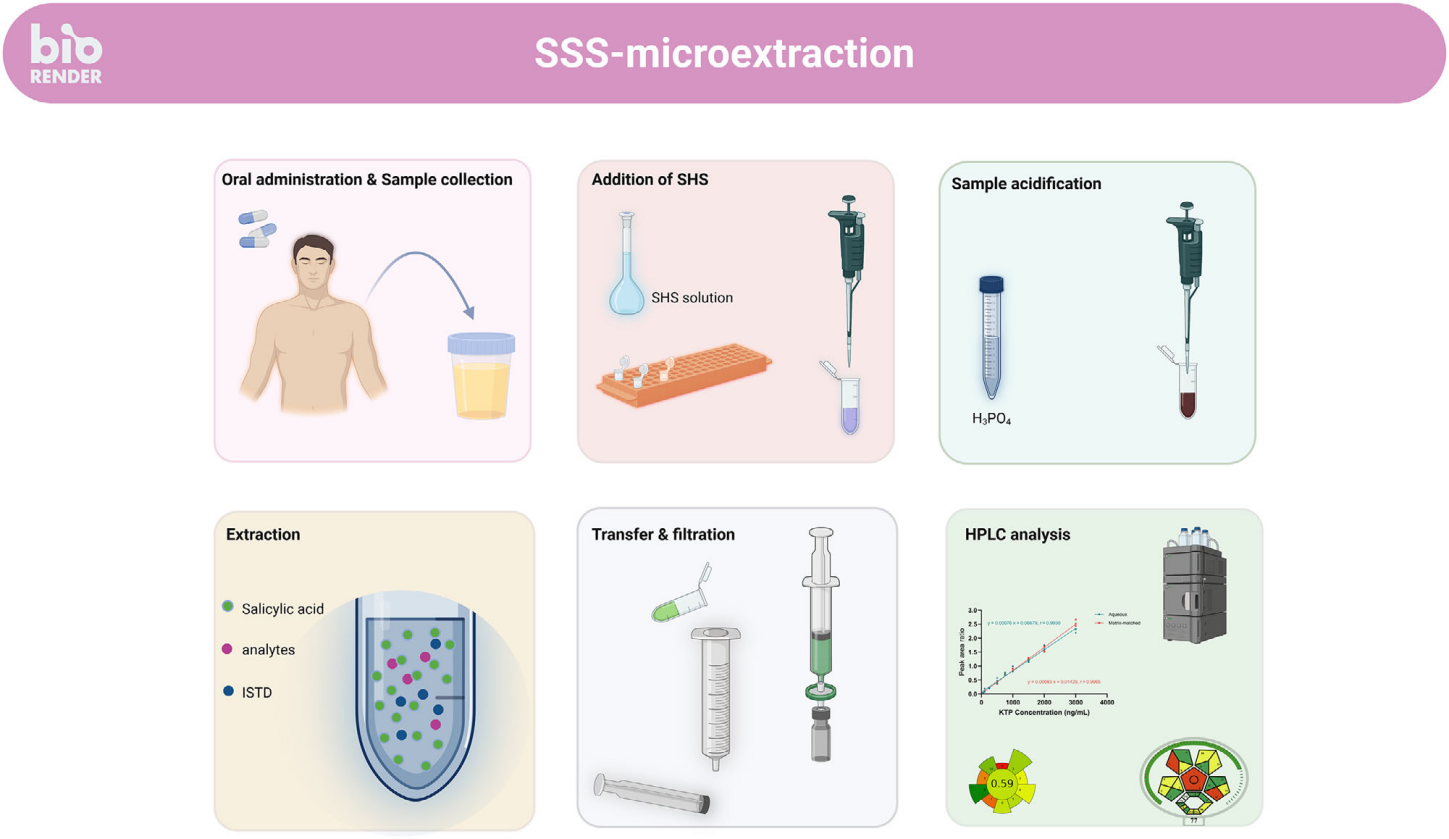
Main steps of the proposed sample preparation procedure. (Created in BioRender by Zacharis, C. (2025) https://BioRender.com/glbs86c.)

Urine samples were obtained from healthy volunteers who had not received any treatment, using sterile containers. All participants were fully informed about the study and gave their written consent. The samples were first centrifuged at 4000 rpm for 10 min and then stored in a refrigerator up to 48 h until further analysis. To prepare the sample, 750 µL of drug‐free urine was mixed with 550 µL of water, 100 µL of the analytes' mixture (or water for blank samples), and 100 µL of ISTD solution (50 µg/mL). The mixture was transferred into a 2 mL Eppendorf tube, followed by the addition of 200 µL of a 0.75 mol/L sodium salicylate aqueous solution. The tube was vortexed for 10 s, and then 50 µL of 10 mol/L H_3_PO_4_ solution was added to induce the formation of water‐insoluble solid salicylic acid, which facilitated drugs' partitioning onto the solid phase. The contents were retrieved using a disposable 3 mL syringe, and 1 mL of water was added to the tube to rinse away any residual sample. The entire mixture was passed through a disposable nylon filter (0.45 µm) to retain the solidified salicylic acid. The syringe plunger was removed, 500 µL of MeOH was added to the barrel, and the plunger was reinserted to dissolve and elute the salicylic acid into an HPLC vial. The main steps of the procedure are illustrated in Figure 1.

### 2.3 HPLC Conditions

A high‐performance liquid chromatography system with UV detection (HPLC‐UV) from Shimadzu (Kyoto, Japan) was used in this study. The system included two LC‐20AD isocratic low‐pressure pumps, a SPD‐20A UV detector, a SIL‐10AD autosampler, and a CBM‐20A system controller. The HPLC was operated using LC Solutions software (version 1.25 SP4). Chromatographic separations were carried out on a BDS C18 analytical column (100 × 4.6 mm, 3 µm) supplied by ThermoScientific. A gradient elution using 0.1% formic acid (mobile phase A) and MeOH (mobile phase B) was utilized for the separation of the analytes at a flow rate of 0.7 mL/min. The initial %B content was 50% v/v, then elevated to 65% at 20 min, kept constant until 22 min, returned to 50% at 23 min, and finally equilibrated until 30 min. All separations were performed at an ambient temperature. The injection volume was 10 µL. All drugs were monitored at 265 nm while the ISTD at 246 nm.

### 2.4 Method Validation

The developed method was validated following the Food and Drug Administration (FDA) guidelines [25]. Selectivity was assessed by analyzing both blank and spiked urine samples. Linearity was evaluated by plotting the analyte peak area ratio against the known concentrations of calibration standards. The matrix effect was determined by comparing the slopes of external and matrix‐matched calibration curves; a slope ratio between 0.8 and 1.2 was considered acceptable, indicating minimal matrix interference. Precision and trueness were examined at four concentration levels, both within a single day and across three consecutive days. These levels included the lower limit of quantitation (LLOQ), as well as low (LQC), medium (MQC), and high (HQC) quality control concentrations. Acceptance criteria required relative standard deviations (%RSD) to be below 15% for QC levels and below 20% for LLOQ. Trueness, expressed as relative recovery (%RR), needed to fall within 85%–115% for QC samples and 80%–120% for LLOQ. LOD was calculated using signal‐to‐noise ratio of 3.

## 3 Results and Discussion

### 3.1 Study of the SSS Type and Concentration

The type of SSS is a crucial parameter that directly influences the extraction performance [26]. An effective SSS should possess high extraction efficiency and exhibit amphiphilic properties, enabling clear separation into hydrophilic and hydrophobic phases. In addition, it should ideally have a relatively high boiling point, allowing it to solidify (without requiring a cooling step) and be easily collected.

**FIGURE 2:**
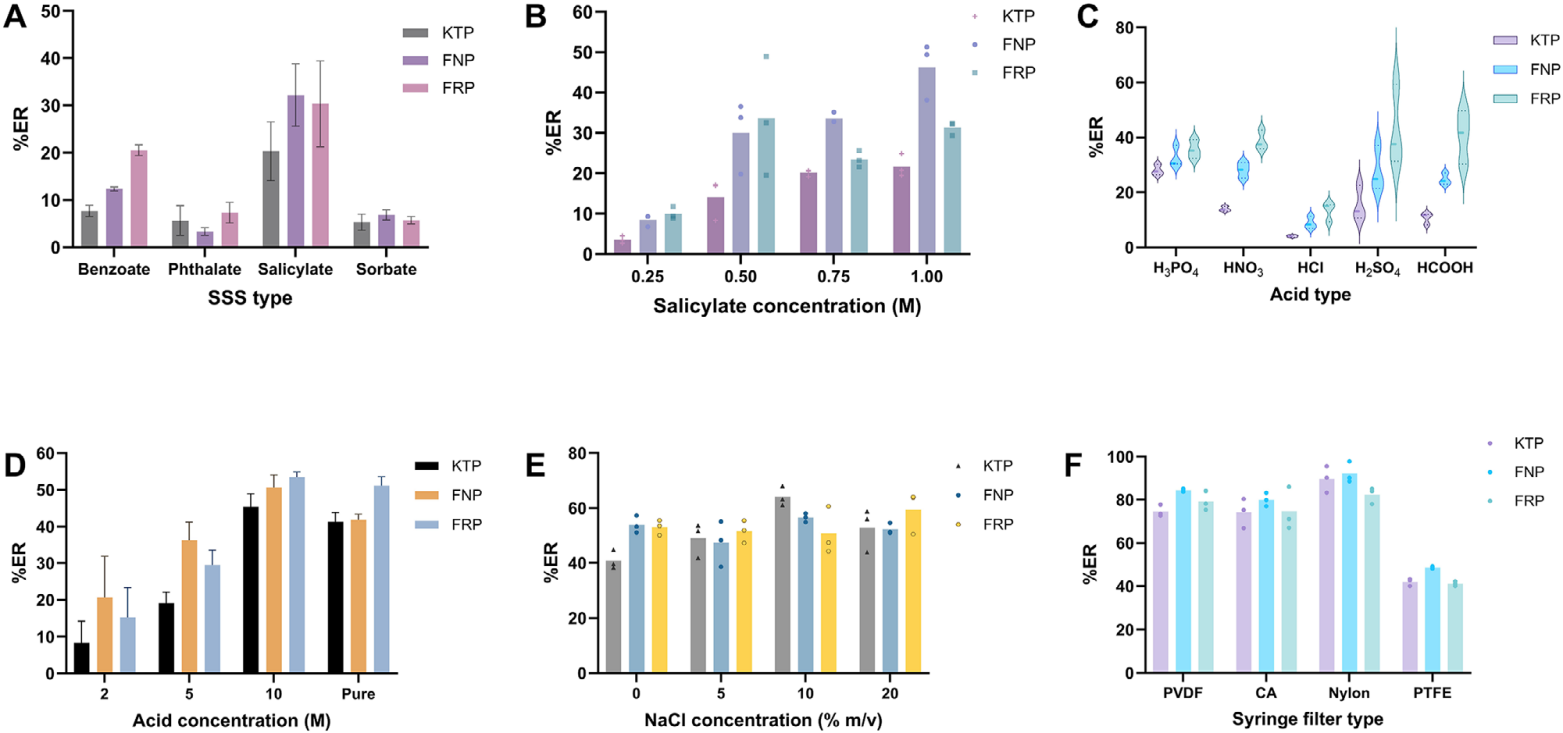
Effect of (A) SSS type, (B) salicylate concentration, (C) acid type, (D) acid concentration, (E) NaCl concentration, and (F) syringe filter type on the %ER of the analytes.

On this basis, four different SSSs were tested including three aromatic and one aliphatic acid salts (sodium salicylic, sodium benzoate, sodium sorbate, and sodium phthalate). The initial experimental conditions included a sample volume of 1000 µL, an aliquot of 200 µL of SSS (0.5 M), and 50 µL of concentrated H_3_PO_4_. The solidified SSS was filtrated though 0.45 µm PTFE syringe filter and dissolved with 1000 µL MeOH. All drugs are acidic with p*K*_a_ ranging between 3.98 and 4.5 [27]. Acidification of the sample enhances the hydrophobicity of the drugs by suppressing the dissociation of carboxylic acid groups (Figure S1). Consequently, the compounds interact via hydrophobic interactions with the aromatic or aliphatic groups of the examined SSSs. As it is shown in Figure 2A, sodium salicylate provided the highest extraction recovery (%ER) values (20%–32%) for all drugs although it exhibited higher variability compared to other SSS tested. Based on these findings, sodium salicylate was selected for further experiments.

The concentration of SSS directly influences both the quantity of the resulting solid phase and the recovery of the analyte. It should be sufficient to enable effective extraction and as low as possible to prevent overloading of the analytical column. The salicylate concentration was tested in the range of 0.25–1 mol/L. Concentrations below 0.1 mol/L were not examined since no visible solidification of salicylic acid was detected. According to Figure 2B, the %ER of the drugs was progressively increased by the salicylate concentration. Similar extraction performances were observed at both SSS concentrations of 0.50 and 0.75 mol/L; however, we chose the latter since it provides better repeatability. The solubility of the 0.75 mol/L salicylic acid in the mobile phase was also tested to avoid potential precipitation of the sample in the HPLC autosampler.

### 3.2 Study of the Acid Type and Concentration

The nature of the acid influences the formation of the water‐insoluble solid salicylic acid, which in turn impacts the efficiency of the extraction process. Strong and weak acids were examined including H_3_PO_4_, HNO_3_, H_2_SO_4_, HCl, and HCOOH. An aliquot of 50 µL of fixed acid concentration (10 mol/L) was used. The pH value of the sample solution after the addition of the acid was approximately 0.5, which was adequate for the quantitative solidification of salicylic acid. Using HCl as a hydrogen donor agent resulted in low %ER values for all analytes, possibly due to the solubilization of solid salicylic acid in the HCl solution. Low %ER values were also observed when using the weak acid HCOOH (Figure 2C). On the contrary, phosphoric acid provided adequate extraction performance (ER: 28%–36%) with low RSD values (< 10%) and was selected for subsequent experiments.

The effect of H_3_PO_4_ concentration was investigated in the range of 2–14.65 mol/L (neat reagent). At lower concentrations, H_3_PO_4_ failed to adequately acidify the salicylic acid phase, leading to reduced extraction efficiency (see Figure 2D). A concentration of 10 mol/L was chosen as an optimal balance between effective extraction and maintaining the analytical column's stability.

### 3.3 Study of the Ionic Strength

Adding salt can lower the solubility of the target compound, promoting its transfer to the solid salicylic acid. On the other hand, increased salt content may raise the sample's viscosity, potentially slowing down analyte diffusion. The sample ionic strength was examined by adding solid NaCl in the range of 0%–20% m/v. As shown in Figure 2E, the extraction efficiency remained almost unaffected in the range examined. Therefore, no salt addition was selected for simplicity.

### 3.4 Study of the Sample and Solvent Volumes

Sample volume plays a key role in determining the method's sensitivity. In this study, its effects were examined over a range of 500–1500 µL. As shown in Figure S2, no significant changes on the %ER of analytes were observed. To achieve the highest preconcentration factors for the analytes, additional experiments were conducted using a sample volume of 1500 µL.

The influence of MeOH volume—used to dissolve salicylic acid after filtration—on the extraction efficiency of the selected drugs was studied within the range of 500–1500 µL. Since solvent volume can influence method's sensitivity, it is preferable to use the minimum amount that still enables effective dissolution of the SSS and the target analytes as well. Figure S3 shows that the %ER remained almost constant in the studied region. As a result, 500 µL was chosen to optimize sensitivity and reduce solvent consumption.

### 3.5 Study of Syringe Filter Type

Four commonly used disposable syringe filter types—PTFE, cellulose acetate (CA), nylon, and PVDF—were evaluated. All these materials are chemically compatible with MeOH. Experimental results showed significantly lower %ERs when using PTFE filters, likely due to nonspecific binding of the analytes to the hydrophobic PTFE surface (Figure 2F). The other filter types exhibited similar performance, allowing filter selection to be based on availability. In this study, nylon filters were chosen for subsequent experiments.

### 3.6 Method Validation

The proposed analytical method was assessed for its selectivity, linearity, trueness, precision, limit of detection (LOD), lower limit of quantitation (LLOQ), and matrix effect to confirm its reliability and validity.

**FIGURE 3:**
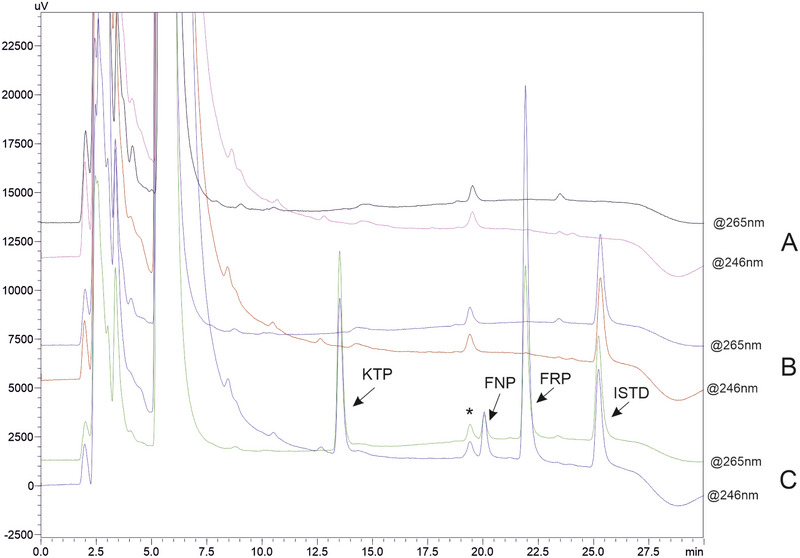
Representative HPLC‐UV chromatograms for the analysis of (A) drug‐free pooled urine sample, (B) drug‐free urine sample spiked with ISTD, and (C) drug‐free urine sample spiked with the analytes (2000 ng/mL) and ISTD, respectively. The peak marked with asterisk (*) corresponded to an impurity present in the sodium salicylate reagent.

The selectivity of the method was evaluated by analyzing both drug‐free pooled urine and samples spiked at a concentration of 2000 ng/mL. As can be seen in Figure 3, the earlier eluted peaks corresponded to the sample matrix and salicylic acid. Moreover, a well‐resolved peak, close to FNP and attributed to a salicylic acid impurity was also detected. No interfering substances were observed in the elution region of the target analyte, demonstrating strong selectivity. Carry‐over was tested by performing three consecutive injections of a spiked sample (3000 ng/mL), followed by the injection of a blank sample. The resulting responses were below 5% of the signals corresponding to the LLOQ levels (50, 100 and 250 ng/mL), confirming effective washing of both the autosampler and the column between injections.

The linearity of the method was assessed using both aqueous and matrix‐matched calibration solutions at a minimum of seven concentration levels: 50–3000 ng/mL for KTP, 250–3000 ng/mL for FNP, and 100–3000 ng/mL for FRP. Each concentration level was tested in triplicate. The calibration curves and their equations are shown in Figure S4 and Table S1. Correlation coefficients exceeded 0.9919, indicating satisfactory linearity. To evaluate parallelism, the slopes of the regression lines were compared. The slope ratios for KTP, FNP, and FRP were 1.09, 0.81, and 0.98, respectively, falling within the acceptable matrix effect range of 0.8–1.2. This confirms that external calibration is suitable for determining analyte concentrations in real urine samples. The LLOQ was 50, 100, and 250 ng/mL for KTP, FRP, and FNP, respectively. At these levels the method demonstrated a trueness within ±20% and a precision (%RSD) of less than 20%. The LOD, based on a signal‐to‐noise ratio of 3, was determined to be 10 ng/mL for KTP and FRP, and 75 ng/mL for FNP.

The method's intraday and interday trueness and precision were evaluated, with the results for human urine samples summarized in Table 1. Intraday precision and trueness were assessed through three replicate analyses conducted on the same day, while interday performance was evaluated over three consecutive days. Precision was reported as the RSD%, and trueness was represented by the RR%. Intraday RSD% values ranged from 0.7% to 12.9%, while interday RSD% values were between 2.3% and 14.3% for all tested drugs. The method's trueness ranged from 82.3% to 105.4% for intraday analysis and from 85.4% to 110.1% for interday analysis across all analytes.

**TABLE 1:** Precision and trueness data of the proposed method for the quantitation of KTP, FNP, and FRP in pooled human urine sample (*n* = 3).

Compound	Concentration level (ng/mL)	Intraday (*n* = 3)		Interday (*n* = 3)	
		Trueness (RR%)**^a^**	Precision (%RSD)	Trueness (RR%)	Precision (%RSD)
KTP	50 (LLOQ)	90.9	11.1	87.0	7.3
	100 (LQC)	91.1	12.9	92.6	14.3
	1000 (MQC)	104.6	5.2	96.8	9.9
	3000 (HQC)	101.3	4.7	103.3	5.7
FNP	250 (LLOQ)	98.0	5.7	98.0	7.7
	500 (LQC)	91.5	3.3	93.2	9.7
	1000 (MQC)	95.6	1.2	100.9	4.6
	3000 (HQC)	105.4	2.0	110.1	6.4
FRP	100 (LLOQ)	82.3	9.0	85.4	4.3
	250 (LQC)	99.9	1.0	98.9	14.2
	1000 (MQC)	104.7	6.2	91.0	11.3
	3000 (HQC)	99.3	0.7	102.1	2.3

^a^ RR: relative recovery.

The robustness of the HPLC separation method was thoroughly examined using a Plackett–Burman design. Eight factorial experiments were designed with the help of TIBCO Statistica 13.3.0 software (TIBCO Software Inc., Palo Alto, CA, USA) to investigate the primary effects of selected variables on the resolution of the analytes, salicylate impurity, and ISTD, as detailed in Table S2. The Pareto chart (Figure S5) revealed that the concentration of FA significantly impacted the resolution between the salicylate impurity/FNP and FRP/FNP pairs. Furthermore, both the flow rate of the mobile phase and the initial %MeOH content were found to have a notable influence on the FRP/FNP resolution (*p* < 0.05). Table S2 shows that the lowest observed resolution (R2) was approximately 1.48, which still meets the acceptable criteria set by the FDA's Center for Drug Evaluation and Research (CDER) [28].

### 3.7 Sample Stability

**FIGURE 4:**
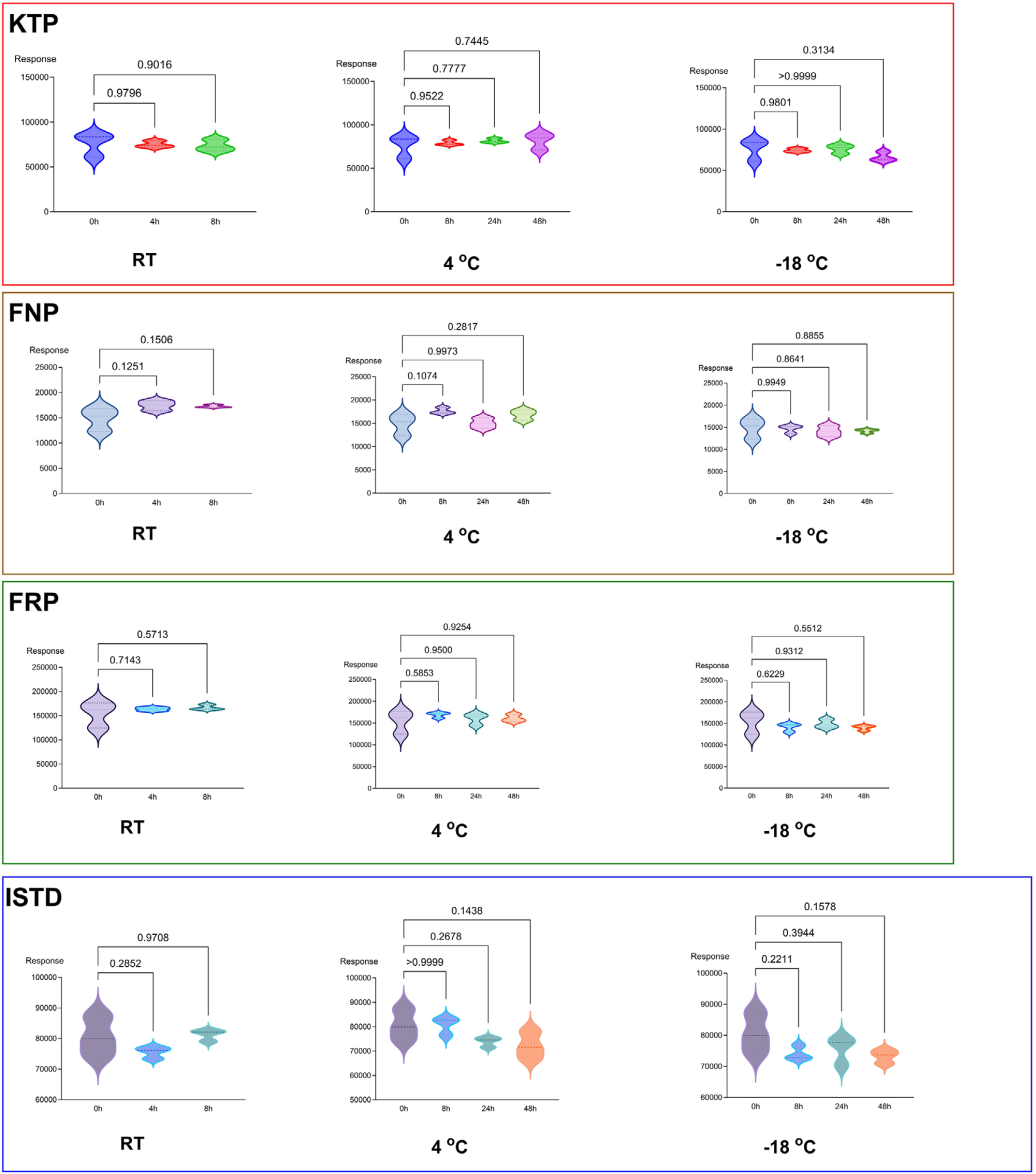
Stability data of the analytes and ISTD in untreated blank urine sample. Numerical values represent the *p* values of the comparison of the peak area of the compound at each time interval with the control sample (*t* = 0 h). RT: room temperature.

The stability of the analytes and ISTD in authentic urine samples was evaluated by analyzing spiked samples (1000 ng/mL) stored under various conditions: at room temperature (0, 4, and 8 h), at +4°C (0, 8, 24, and 48 h), and at −18°C (0, 8, 24, and 48 h). No statistically significant degradation was observed (Figure 4). These findings are consistent with previously reported results [14].

### 3.8 Analysis of Human Urine Samples

The applicability of the proposed analytical scheme was demonstrated by analyzing human urine samples from a patient collected at 0.5, 1, 2, 4, and 8 h after oral administration of KTP‐containing pharmaceutical formulation (OKITASK 25 mg/tab). Representative chromatograms from the urine analysis are illustrated in Figure S6. Three individual extractions of each sample were carried out. The KTP concentration in the samples ranged from 125 to 1900 ng/mL.

### 3.9 Comparison With Other Published Bioanalytical Approaches

The proposed SSS‐based microextraction approach was evaluated against previously published approaches for the analytes determination in biological samples [14, 15, 16, 17, 18, 21, 22, 24].

In comparison to other techniques, the developed method offers a significant advantage by eliminating the need for solid‐phase extractants such as membranes, capsules, advanced materials (e.g., COFs and MOFs), or cartridges, thereby presenting a more cost‐effective alternative. The SHS‐based method [24] achieves extraction within just 30 s, which is considerably faster than FPSE, membrane‐based, and MOF‐functionalized monolith approaches, enabling higher sample throughput. However, this method requires relatively large volumes of organic solvents, including 4 mL of acetonitrile and 500 µL of *N*,*N*‐dimethylcyclohexylamine as the extraction solvent, making it less favorable from a green analytical chemistry (GAC) perspective. As shown in Table 2, the proposed method demonstrated satisfactory precision and accuracy, although its sensitivity was lower compared to other microextraction‐based techniques.

**TABLE 2:** Comparison of the proposed method with previously published bioanalytical HPLC approaches.

Sample	Sample pretreatment	Extraction time	LOQ (ng/mL)	RSD (%)	RR (%)	Ref.
Urine	FPSE	45 min	25	< 10.2	82.9–101.6	[14]
Saliva	FPSE	10 min	80	< 7.0	85.6–112.7	[15]
Serum	COF	46 min	20	< 15.3	82.0–118	[16]
Urine	MOF‐functionalized monolith	240 min	0.7–20.5	< 14.0	71.0–78.0	[17]
Urine	HF membrane‐based liquid phase microextraction	100 min	20–100	< 20.7	92.5–126.9	[18]
Urine	Automated electric‐field‐driven liquid phase microextraction	10 min	500 (KTP)	< 5.4	52–77	[19]
Plasma	MOF‐functionalized SPE	15 min	1	< 5.5	65.5–98.9	[21]
Urine	SDME	10 min	25	< 3.6	80	[22]
Saliva, urine	SHS	30 s	220 (KTP)	< 3.7	95.7–109.2	[24]
**Urine**	**SSS**	**30 s**	**50–250**	**< 14.3**	**85.4–110.1**	**Proposed method**

### 3.10 Assessment of Method Greenness

The method's environmental friendliness was evaluated using four tools: the modified ComplexGAPI (ComplexMoGAPI), AGREEprep, “Click Analytical Chemistry” index (CACI), and Analytical GREEnness (AGREE) [29, 30, 31, 32].

**FIGURE 5 (:**
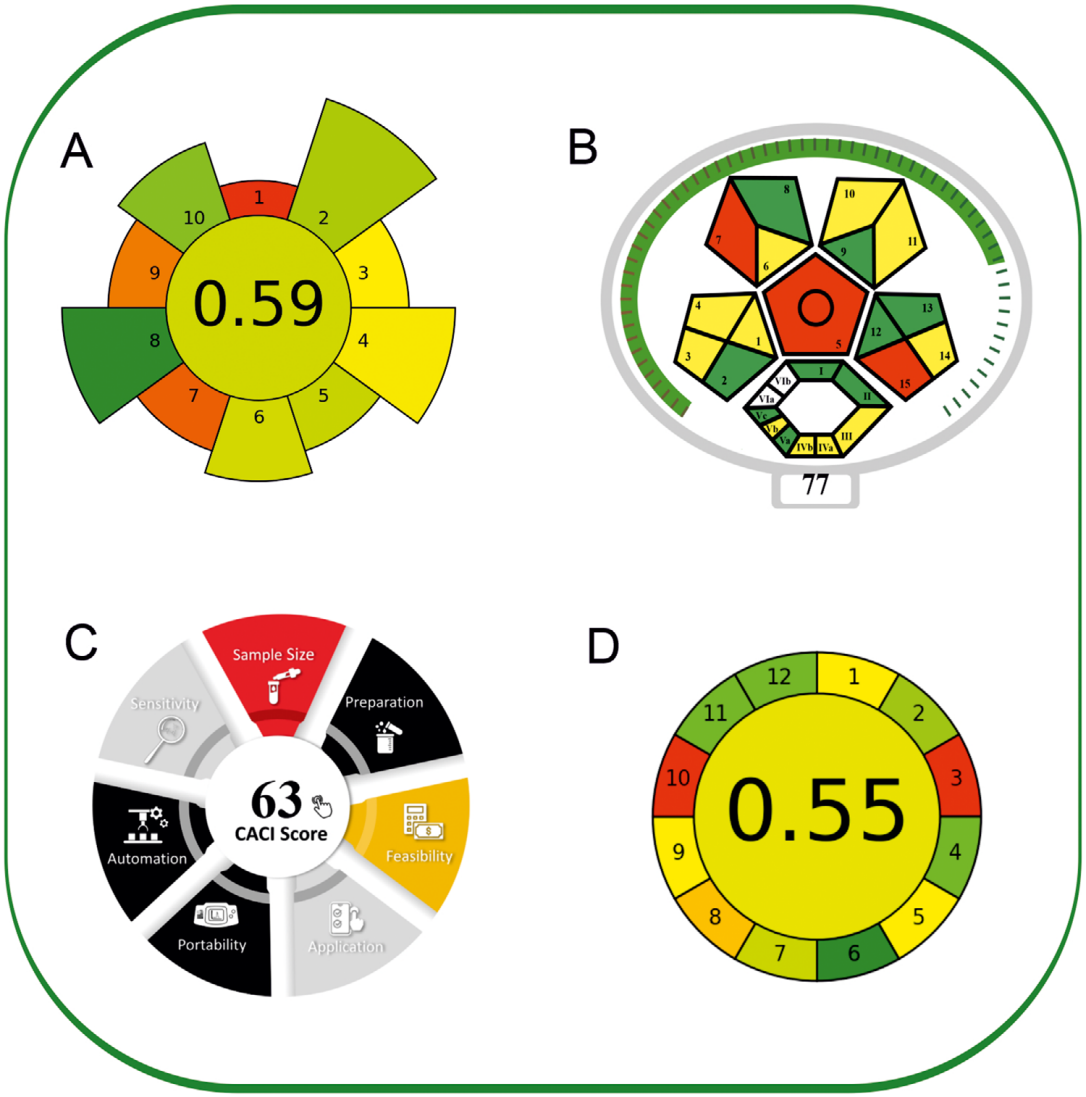
A) AGREEprep, (B) ComplexMoGAPI, (C) CACI, and (D) AGREE scores for the developed microextraction method.

AGREEprep is a tool tailored to assess the environmental impact of sample preparation steps. It uses a colored pictogram with a central numerical score ranging from 0 (indicating poor environmental performance) to 1 (representing ideal performance or no sample preparation required) [29]. The central circle's color and score indicate the overall environmental friendliness of the sample preparation process. Around it, 10 segments each correspond to a distinct performance criterion. Their lengths reflect the default assigned importance of each criterion, while their colors visually convey how well each aspect performs. Τhe overall score of AGREEprep assessment was found to be 0.59 (Figure 5A). Notably, Criterion 2 scored well due to the absence of highly toxic reagents. However, three criteria were below 0.5: sample preparation location (Criterion 1), integration and automation (Criterion 7), and post‐sample preparation configuration (Criterion 9).

ComplexMoGAPI is an advanced version of ComplexGAPI that combines visual representations with precise scoring to better assess the environmental impact of analytical methods. It uses a detailed scoring system ranging from 0 to 100. Based on the experimental data, the method achieved a cumulative greenness score of 77, reflecting a relatively high level of environmental sustainability (Figure 5B).

CACI is a newly developed, practical, and efficient tool for assessing analytical methods, inspired by the straightforward and dependable nature of click chemistry [31]. This tool evaluates essential aspects such as sample size, preparation, feasibility, applicability, portability, sensitivity, and level of automation. A color‐coded pictogram visually represents the performance: colored indicates high performance, gray shows moderate performance, and black signifies poor performance or failure to meet the standards (Figure 5C).

The AGREE tool employs a circular chart where each segment represents one of the (GAC) principles, with scores ranging from 0 (not green) to 1 (fully green) [32]. The resulting diagram (Figure 5D) showed a promising score of 0.55, indicating strong environmental compatibility. The AGREE assessment highlighted some drawbacks, including off‐line analysis (Principles 1 and 3) and high energy use associated with the liquid chromatography system (Principle 9). Nevertheless, it also emphasized several benefits, such as minimal sample volume (Principle 2), simplified sample preparation (Principle 4), and the exclusion of derivatization reagents (Principle 6). Furthermore, the method excelled in terms of solvent safety and efficient use (Principles 11 and 12). Overall, the findings suggest that the developed method supports GAC principles and minimizes environmental impact.

## 4 Conclusions

In this study, we introduced a novel LPME method utilizing salicylic acid for the detection of KTP, FNP, and FRP in human urine. The technique leverages a pH‐induced phase transition of salicylic acid, which enables efficient dispersion and phase separation in a single step. The solidified salicylic acid was easily collected using a syringe filter. The extensive surface area of the solidified solvent interacting with the sample facilitated efficient extraction of the target analytes. This new approach offers notable advantages, including simplicity, speed, cost‐efficiency, and the use of environmentally friendly solvents. The non‐automated nature of the proposed approach could be considered a limitation; however, the ability to process samples in parallel can help balance this drawback by improving overall throughput. The method demonstrated good linearity, trueness, and precision across the validated concentration range and lower LODs (10–75 ng/mL) compared to previously published SHS‐based approaches (40–180 ng/mL) [24]. Its practical application was confirmed by successfully analyzing real urine samples from a patient following oral administration of a KTP‐containing medication.

## Author Contributions

**Christina Patakidou**: Formal analysis, Data curation, Investigation, Validation, **Marianna Ntorkou**: Investigation, Data curation **Constantinos K. Zacharis**: Conceptualization, Methodology, Supervision, Visualization, Writing – Original Draft, Writing – Review & Editing.

## Conflicts of Interest

The authors declare no conflicts of interest.

## Supporting information




**Supporting Information file 1**: jssc70223‐sup‐0001‐SuppMat.docx
