# Supplementary material for: Sustainable Microextraction Using Switchable Solubility Solvent for the Liquid Chromatographic Determination of Three Profenoid Drugs in Urine Samples
Source: J Sep Sci. 2025 Jul 12;48(7):e70223. doi: 10.1002/jssc.70223 (PMC12254904; doi:10.1002/jssc.70223)
Supplement: Supplementary file 1 — Supporting Information file 1: jssc70223‐sup‐0001‐SuppMat.docx [file JSSC-48-e70223-s001.docx]

**Supplementary material**

**Sustainable microextraction using switchable solubility solvent for the liquid chromatographic determination of three profenoid drugs in urine samples**

Christina Patakidou^1,2^, Marianna Ntorkou^1^, Constantinos K. Zacharis*^1^

*^1^Laboratory of Pharmaceutical Analysis, Department of Pharmacy, Aristotle University of Thessaloniki, 54124 Thessaloniki, Greece;*

*^2^Laboratory of Analytical Chemistry, Department of Chemistry, Aristotle University of Thessaloniki, 54124 Thessaloniki, Greece;*

|  |  |
| --- | --- |

*Corresponding author

Constantinos K. Zacharis

Associate Professor

Laboratory of Pharmaceutical Analysis, School of Pharmacy,

Aristotle University of Thessaloniki (AUTh),

GR-54124, Greece

Tel: +30 2310997663

E-mail: czacharis@pharm.auth.gr

**
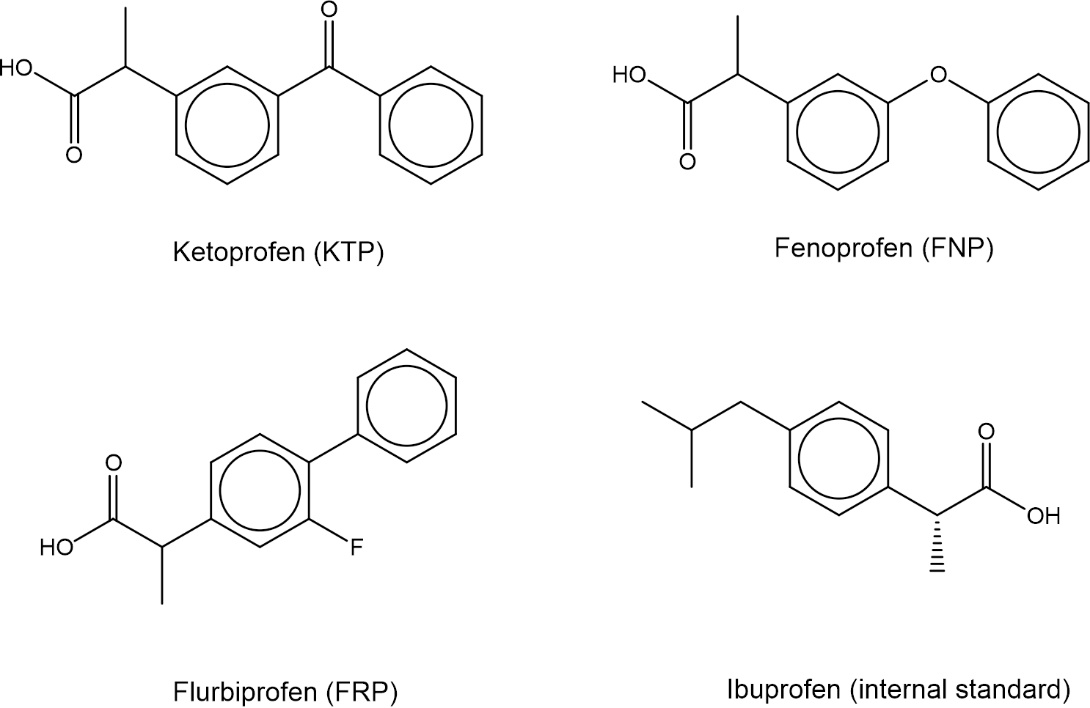
**

**Figure S1.** Chemical structures of ketoprofen, fenoprofen, flurbiprofen and ibuprofen (internal standard).


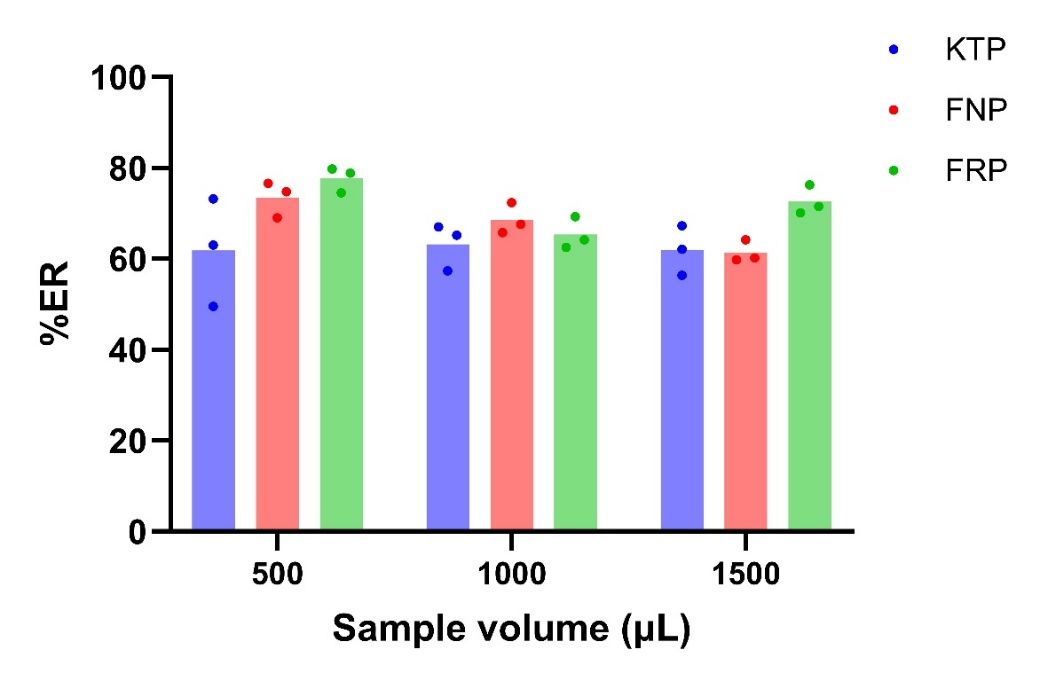


**Figure S2.** Effect of the sample volume on the %ER of the analytes.


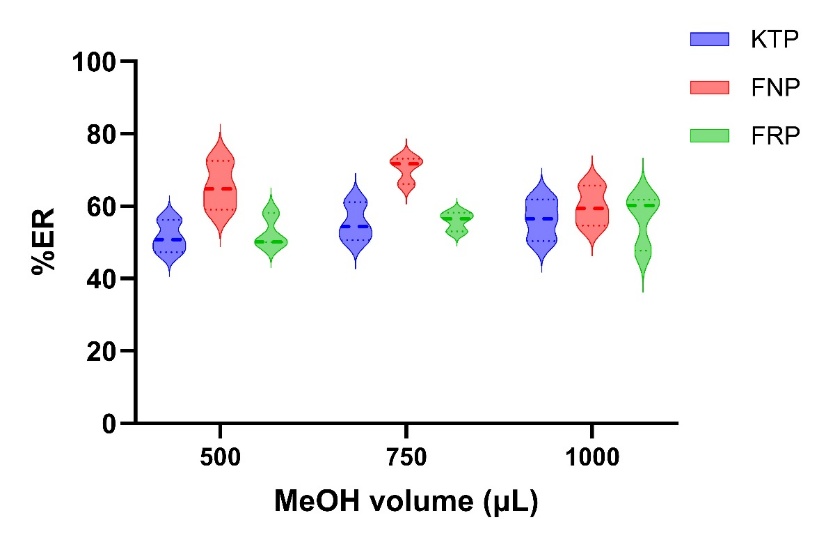


**Figure S3.** Effect of the methanol volume on the %ER of the analytes.


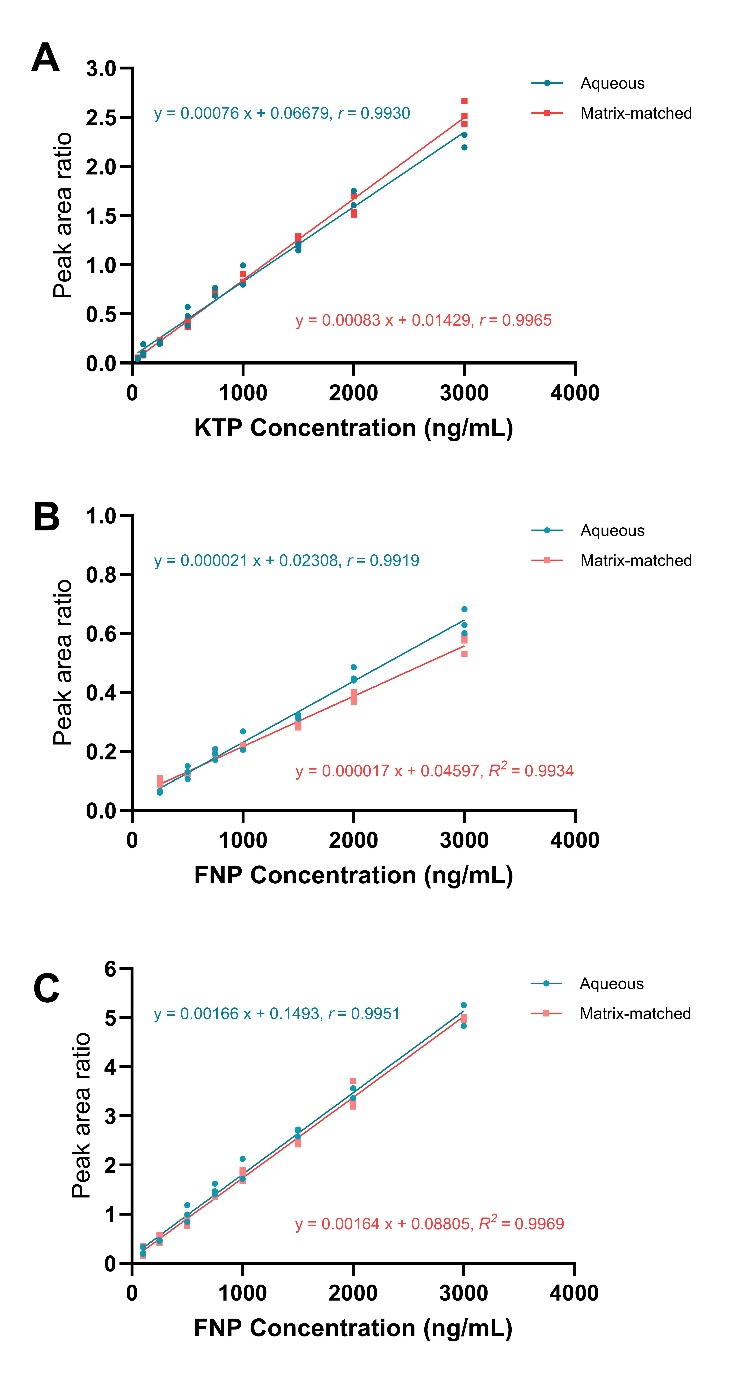


**Figure S4.** Aqueous and matrix-matched calibration curves of A) KTP, B) FNP and C) FRP.


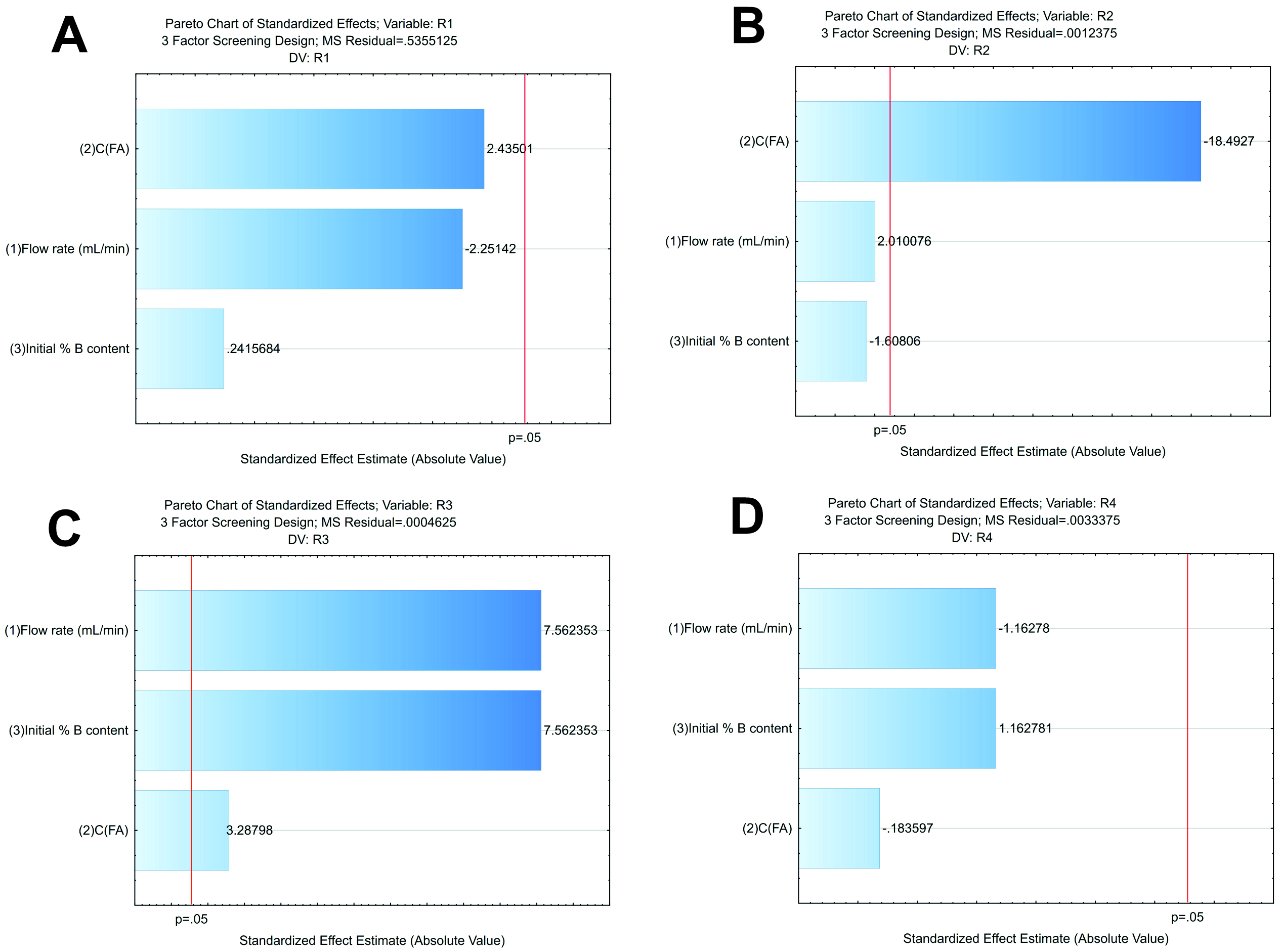


**Figure S5**. Pareto charts for the robustness test of HPLC parameters. Responses: A) R1 (salicylate impurity-KTP), Β) R2 (FNP-salicylate impurity), C) R3 (FRP-FNP) and D) R4 (ISTD-FRP).


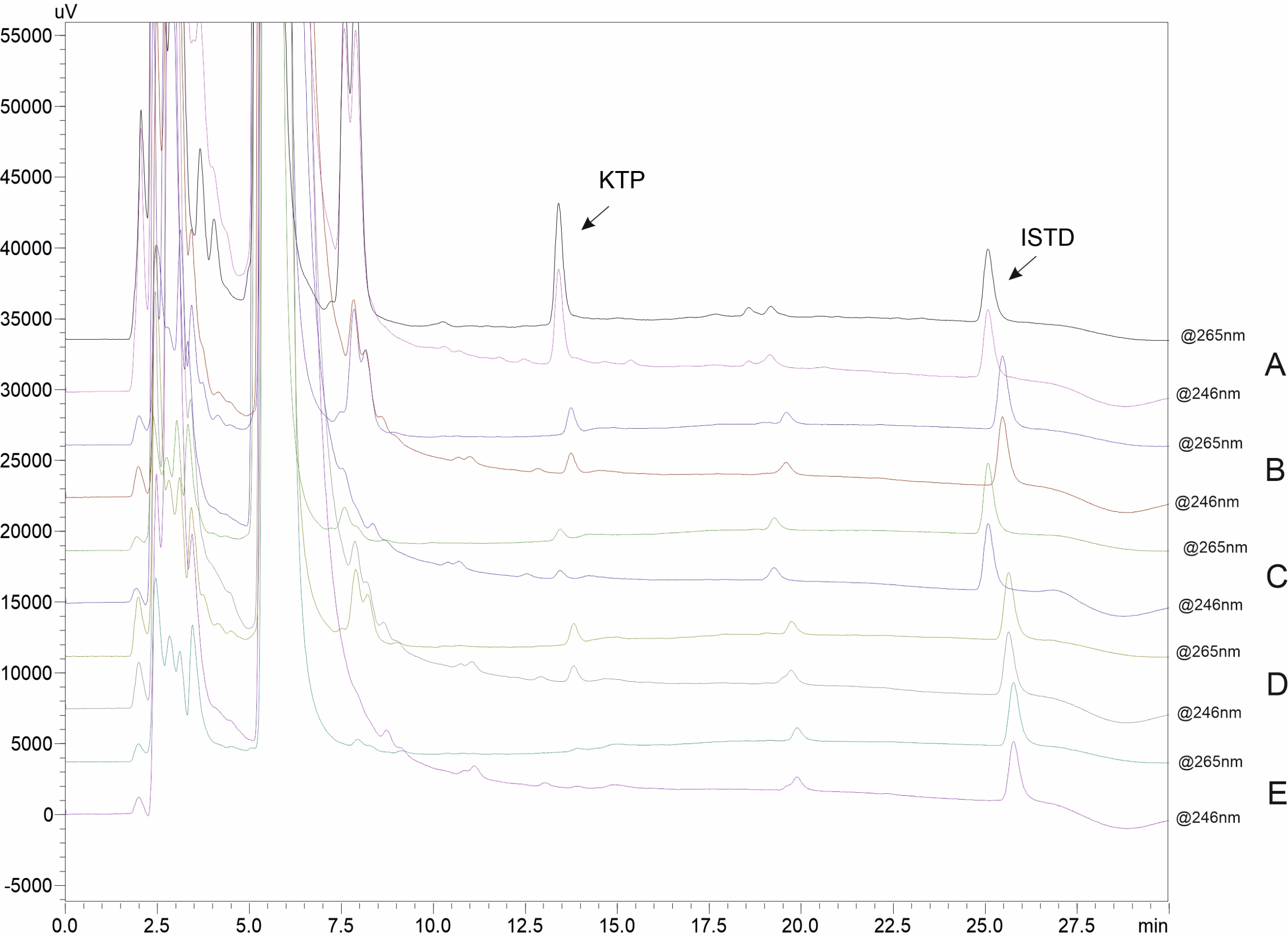


**Figure S6.** Representative HPLC chromatograms from the analysis of KTP collected at A) 0.5h, B) 1h, C) 2h, D) 4h and E) 8h after administration of a KTP-containing formulation (OKITASK® 25 mg/tab).

**Table S1.** Unweighted regression equations, dynamic linear range and determination coefficients for the analyte of interest.

| **Analyte** | **Dynamic linear range (ng/mL)** | **Regression equation**  ***R*^1^ = (A±SD_A_) × *C* + (B±SD_B_)** | ***r*** |
| --- | --- | --- | --- |
| KTP | 50 – 3000 | *R* = (76**±**1.9)×10^-5^ × C + (0.067±0.024) | 0.9930 |
| FNP | 250 – 3000 | *R* = (20**±**0.6)×10^-5^ × C + (0.023±0.001) | 0.9919 |
| FRP | 100 - 3000 | *R* = (166**±**3.7)×10^-5^ × C + (0.149±0.050) | 0.9951 |

^1^R: analyte-to-ISTD peak area ratio, A: slope, SD_A_: standard deviation of slope, B: intercept, SD_B_: standard deviation of intercept

**Table S2.**  Plackett-Burman design for the evaluation of the robustness of HPLC separation.

| **Run No** | **Flow rate (mL/min)** | **C(FA)** | **Initial %B content** | **Dummy1** | **Dummy2** | **Dummy3** | **Dummy4** | **R1^a^** | **R2^b^** | **R3^c^** | **R4^d^** |
| --- | --- | --- | --- | --- | --- | --- | --- | --- | --- | --- | --- |
| 3 | 0.65 | 0.11 | 49 | -1 | 1 | -1 | 1 | 13.19 | 1.52 | 4.51 | 7.17 |
| 6 | 0.75 | 0.09 | 51 | -1 | 1 | -1 | -1 | 10.58 | 1.99 | 4.7 | 7.2 |
| 2 | 0.75 | 0.09 | 49 | -1 | -1 | 1 | 1 | 11.56 | 2.03 | 4.59 | 7.21 |
| 5 | 0.65 | 0.09 | 51 | 1 | -1 | -1 | 1 | 13.3 | 1.91 | 4.55 | 7.29 |
| 1 | 0.65 | 0.09 | 49 | 1 | 1 | 1 | -1 | 12.79 | 2.02 | 4.47 | 7.19 |
| 7 | 0.65 | 0.11 | 51 | -1 | -1 | 1 | -1 | 13.8 | 1.48 | 4.65 | 7.32 |
| 8 | 0.75 | 0.11 | 51 | 1 | 1 | 1 | 1 | 13.32 | 1.57 | 4.74 | 7.16 |
| 4 | 0.75 | 0.11 | 49 | 1 | -1 | -1 | -1 | 12.96 | 1.54 | 4.61 | 7.21 |

^a^R1: Resolution of salicylate’s impurity-KTP

^b^R2: Resolution of FNP-salicylate impurity

^c^R3: Resolution of FRP-FNP

^d^R4: Resolution of ISTD-FRP
